# RELMα establishes a permissive environment for influenza virus infection through direct effects on lung epithelial cells

**DOI:** 10.1128/jvi.02225-25

**Published:** 2026-07-16

**Authors:** Roksana Shirazi, Sang Yong Kim, Aaron Cruz, James P. Stumpff, Stefanie N. Sveiven, Sumaya Alaama, Jennell Jennett, Brandon H. Le, Meera G. Nair, Juliet Morrison

**Affiliations:** 1 Department of Microbiology and Plant Pathology, University of California, Riverside 207030, Riverside, California, USA; 2 Division of Biomedical Sciences, School of Medicine, University of California, Riverside 117249, Riverside, California, USA; 3 Institute for Integrative Genome Biology, Bioinformatics Core Facility, Department of Botany and Plant Sciences, University of California, Riverside 8790, Riverside, California, USA; Emory University School of Medicine, Atlanta, Georgia, USA

**Keywords:** epithelial cells, Retnla, RELMα, influenza virus

## Abstract

Beyond a physical barrier, the lung epithelium provides an essential line of defense against infectious pathogens, such as influenza A viruses (IAVs), by producing antimicrobial factors and triggering innate and adaptive immunity. On the other hand, IAV infects lung epithelial cells and manipulates their function to establish a non-protective, permissive environment. Identification of epithelial factors and pathways that can be targeted to restore optimal immunity would provide new therapeutic options against IAV. Here, we demonstrate an unexpected non-immune function for the cytokine RELMɑ in mediating a permissive environment for IAV infection through lung epithelial cell-intrinsic effects. Murine infection with IAV A/California/04/2009 (H1N1) led to significant RELMɑ secretion by IAV-infected EpCAM+ epithelial cells. Both whole-body and club cell-specific RELMɑ-deficient mice (*Retnla*^−/−^ and *Retnla*^ΔCC10^) had significantly reduced infectious virus, specifically in epithelial cells, whereas the lung immune response was unaffected. RELMɑ treatment increased IAV infection of murine lung epithelial cell lines, and transcriptomic analysis indicated that RELMɑ-induced permissiveness to IAV was associated with cell-intrinsic changes in metabolic activity. Collectively, these studies suggest a direct non-immune role for RELMɑ in mediating a virus-permissive environment in epithelial cells.

**IMPORTANCE:**

Seasonal influenza causes over 3 million cases of severe disease and an estimated 500,000 deaths worldwide annually, highlighting the need for new therapeutic targets. The host response to infection is a significant factor in determining influenza outcome and can be subverted by the virus. The epithelial barrier is the first line of defense and a target for influenza A viruses (IAVs), which seek to establish a permissive environment for their own growth, and these pathways could be targeted to control viral burden. Here, we identify RELMɑ, a new host factor that is induced in epithelial cells upon IAV infection and aids viral infection. Through RELMɑ-deficient mouse models, lung epithelial cell cultures, and gene expression analysis, we show that RELMɑ acts directly on epithelial cells to change cell metabolic pathways and promote viral growth. These findings provide a unique epithelial cell protein that can be inhibited to limit influenza infection.

## INTRODUCTION

Influenza virus infection is a global health concern, causing 290,000–650,000 deaths every year globally due to respiratory disease (1). Influenza A virus (IAV) infection results in a wide range of symptoms ranging from mild respiratory disease to severe pneumonia, acute respiratory distress syndrome, and even death (2–4). The IAV genome consists of eight negative-sense RNA segments, which encode the viral structural and non-structural proteins. IAV enters host cells by binding to sialic acids linked to cell surface galactose on glycoproteins or glycolipids through hemagglutinin (HA) (5, 6). HA is the primary target of current influenza virus vaccines, but other viral components like neuraminidase (NA) and matrix (M) are also targets for vaccine development (7–9). The constant evolution of IAV strains due to antigenic drift and shift requires the development of yearly seasonal influenza vaccines. This time-consuming and expensive process sometimes results in mismatches between the vaccines and the circulating strains. For example, during the 2019–2020 influenza season, the protection conferred by the vaccine against H1N1 virus was lower than in previous years because the circulating H1N1 virus that emerged that season had accumulated unexpected changes due to antigenic drift (10). Identifying host factors that regulate viral replication and/or tissue damage during IAV infection could pinpoint new targets for the development of influenza therapeutics.

Epithelial cells of the upper and lower respiratory tract, such as ciliated cells, club cells, and type I and II alveolar epithelial cells, are the primary targets of IAV infection (11–13), such that they have developed several defense mechanisms against IAV, including the production of interferon, antiviral proteins, and chemokines that recruit immune cells for viral killing but can cause tissue-damaging inflammation. IAV infection also triggers apoptosis of epithelial cells to limit further virus propagation and proinflammatory cytokine production (14–16). Nonetheless, these epithelial cell defense mechanisms can be countered by the virus, including subverting the epithelial response to its own benefit. These subversion mechanisms are less well understood, and identification of new host epithelial cell-derived proteins that counter antiviral immunity may uncover new host-targeted therapeutics.

RELMα (resistin-like molecule alpha), also known as FIZZ1 (found in inflammatory zone) and HIMF (hypoxia-induced mitogenic factor), encoded by the *Retnla* gene, is a small, secreted protein that was first identified in a murine asthma model (17, 18). RELMα belongs to the RELM family of proteins that include human resistin and RELMβ, which are expressed in inflammatory diseases (19, 20). Since its discovery, RELMα has been shown to have remarkable pleiotropy in function based on the context and cell type, from inducing stromal cell proliferation and fibrosis and regulating immune cell activation in type II immune responses and hypoxia to promoting metabolic homeostasis (21–29). RELMα is expressed by non-immune cells, such as lung epithelial cells, and immune cells, such as macrophages, which may dictate its functional outcome. Factors that induce RELMα include T-helper type II cytokines, such as IL-4, as well as hypoxia and oncostatin M (OSM) (30–32). RELMα expression is also induced in tissue injury and upon phagocytosis of apoptotic cells. Given that influenza infection causes lung tissue injury, cell apoptosis, and hypoxia, the role of this study was to investigate the induction and function of RELMα in IAV infection. Wild-type (WT) and RELMα-deficient mice were infected with IAV, and RELMα expression was quantified. Additionally, the function of RELMα on infection outcomes, including the lung inflammatory response and viral burden, was determined using whole-body and lung epithelial cell-specific RELMα-deficient mice. *In vitro* infection of murine lung epithelial cell lines was also performed to determine direct effects of RELMα on epithelial cells. Overall, results from this study demonstrate that IAV infection induces lung epithelial cell-specific RELMα expression that negatively impacts the host’s ability to eliminate the virus.

## MATERIALS AND METHODS

### Mice

*Retnla*-deficient transgenic mice were generated by genOway by targeting *Retnla* exons 2–4 using Cre recombinase and Flp-mediated excision and replacement with the tDT reporter gene. Briefly, mice were bred with a genOway proprietary Cre deleter C57BL/6 mouse line to generate constitutive *Retnla*^tDT/+^ mice. *Retnla*^tDT/+^ heterozygote mice were crossed with C57BL/6J mice to generate littermate homozygote (tDT/tDT or knockout [KO]) and wild-type (+/+) mice after two generations, then bred in-house. Mice were genotyped by PCR and RELMα serum ELISA. B6N.129S6(Cg)-Scgb1a1tm1(Cre/ERT)Blh/J mice were purchased from the Jackson Laboratory and crossed with *Retnla*^Flox/Flox^ mice to generate CC10 Cre- and CC10 Cre+ *Retnla*^Flox/Flox^ littermates as previously described and validated (29). Mice were age-matched (8–14 weeks old), sex-matched for experiments, and housed five per cage under an ambient temperature with a 12-h light/12-h dark cycle. Mice were challenged with intranasally delivered PBS or 10² PFU (for assessment at day 9) and 10³ PFU (for assessment at day 3) of IAV to evaluate immune responses at different stages of infection, ensuring measurable early responses at day 3 with a higher dose and allowing a prolonged immune response to be observed at day 9 with a lower dose. Lungs were collected to allow each lung to be used for separate endpoints. The main bronchus on one side was tied off by suture and excised for the viral plaque assay, leaving the other lung for inflation and histological assessment.

### Viruses

A/California/04/2009 H1N1 virus was a kind gift from Adolfo García-Sastre (Icahn School of Medicine at Mount Sinai School) and Venus-expressing A/California/04/2009 H1N1 virus was a kind gift from Luis Martinez Sobrido (Texas Biomedical Research Institute) (33). Virus stocks were propagated in specific-pathogen-free eggs (Charles River Laboratories) and titrated by plaque assays on MDCK (NBL-2) cells from ATCC.

### Tamoxifen injection

Tamoxifen was prepared at a concentration of 20 mg/mL in corn oil and stored frozen until use. Mice were injected intraperitoneally with 100 µL of tamoxifen solution every other day for a total of five injections. Mice were given seven full days to recover before the start of influenza virus infection.

### Cytokine quantification

For sandwich ELISA, RELMα capture and biotinylated detection antibodies were used according to previously described protocols (29), with a standard curve of recombinant RELMα based on the manufacturer’s recommendation (top 2 ng/mL). Briefly, recombinant murine RELMα protein, rabbit antimurine RELMα, and biotinylated rabbit antimurine RELMα were purchased from PeproTech (cat #450-26, 500-P214, and 500-P214BT, respectively). The plates were coated overnight at 4°C with rabbit antimurine RELMα at a concentration of 0.5 µg/mL, and the ELISA was completed on the following day. A detection antibody was also used at a concentration of 0.5 µg/mL. Cytometric bead array was conducted with LEGENDplex Mouse Inflammation Panel based on the manufacturer’s instructions (BioLegend).

### Immunofluorescence staining

The right lobe of mouse lung was inflated with 400 μL of OCT and 4% PFA/30% sucrose PBS (2:1) mixture and stored overnight in 4% PFA/30% sucrose PBS at 4°C. After 24 h, tissue was removed from the solution and incubated for 24 h in 30% sucrose PBS. Lungs were blocked in OCT, sectioned at 10 µm, and stained by hematoxylin and eosin (H&E). For immunofluorescent (IF) staining, sections were incubated with rabbit anti-RELMα (Peprotech), anti-NS1 (Invitrogen), CC10 (Invitrogen), rat APC-conjugated anti-RELMα (eBioscience), goat anti-IAV (Millipore Sigma), anti-CC10 antibodies (Santa Cruz Biotechnology), and *Griffonia simplicifolia* lectin I (Vector Laboratories) overnight at 4°C. Sections were incubated with fluorochrome-conjugated secondary antibodies for 2 h at 4°C and then counterstained with DAPI. Multiple images per biological replicate were taken by DM5500B (Leica) or BZ-X800 (Keyence) and then counted by QuPath 0.2.3. Biological replicates are presented in the figure panels (34).

### Bronchoalveolar lavage wash and flow cytometry

A catheter (20G Insyte Autoguard Shielded IV catheter; BD, cat #381537) was placed through a small incision in the trachea and secured by suture wire. Bronchoalveolar lavage fluid (BALF) and cells were recovered by filling the lungs and removing 800 μL of ice-cold 1× PBS. Cells were recovered by centrifugation, and supernatant was collected for ELISA and cytometric bead array. Leukocytes were enumerated by an automated cell counter (DeNOVIX) for flow cytometry and stained with Zombie Aqua Fixable Viability Kit (BioLegend). Then, cells were blocked with 0.6 μg rat IgG and 0.6 μg anti-CD16/32 (93, BioLegend), followed by staining for 30 min with antibodies for MerTK (2B10C42), MHCII (M5/114.15.2), CD11b (M1/70), Ly6G (1A8), CD11c (N418), F4/80 (BM8) (BioLegend), and SiglecF (E50-2440) (BD Biosciences). Cells were then washed and analyzed on an LSRII (BD Biosciences) or Novocyte (Agilent) instrument, followed by data analysis using FlowJo v10.8.1 (Tree Star Inc). Cells in all animals from each group were randomly chosen by the downsample plugin in FlowJo and concatenated to one file. Uniform manifold approximation and projection (UMAP) plots were generated with the concatenated file by a UMAP plugin in FlowJo.

### Plaque assay

The left lobe of the mouse lung was homogenized in 1 mL PBS. MDCK (NBL-2) cells from ATCC were seeded in six-well culture plates and incubated at 37°C in 5% CO_2_ for 24 h. Tenfold serial dilutions of lung homogenates were prepared in a solution containing 1× PBS, 0.21% BSA, 1% Pen/Strep, and 1% Ca/Mg. Cells were washed once with PBS before adding 200 μL of the serial dilutions. Plates were incubated at 37°C and were rocked side-to-side and forward-to-back every 15 min to distribute virus inoculum over the monolayer of cells for 1 h. TPCK-treated trypsin (1 mg/mL) was added at a 1:1,000 ratio to a supplemented 2× DMEM (2× DMEM, 2% Pen/Strep, 0.42% BSA, 20 mM HEPES, 0.24% NaHCO_3_, 0.02% DEAE-dextran) before mixing with 1.5% Oxoid Agar at a 1:1 ratio. Two milliliters of the agar overlay was added to each well and allowed to cool for 15 min at room temperature before transferring to a 37°C incubator. Plates were incubated for 96 h. After incubation, plates were fixed with 1 mL of a 3.7% formaldehyde solution and incubated for 10 min to neutralize infectious virus. The overlay was removed with a spatula, and the cells were stained for 20 min with crystal violet (0.095% crystal violet, 2.8% ethanol, and 19% methanol). Plates were rinsed in tap water, and plaques were counted to determine viral titers.

### MLE-12 cells

MLE-12 cells were cultured in HITES Media: DMEM/F-12 with HEPES, 1× insulin-transferrin-selenium, 2% FBS, 100 U/mL penicillin-streptomycin, 2 mM L-glutamine (Gibco), 10 nM hydrocortisone, and 10 nM β-estradiol (Sigma-Aldrich) were added to media according to the standard ATCC protocol for MLE-12 cells to maintain their phenotype and kept at 37°C in 5% CO_2_. MLE-12 cells were pre-treated with RELMα and infected with 0.01 or 10 MOI of Venus-expressing pH1N1 A/California/04/2009 viruses. At indicated time points, the supernatant was removed, and cells were stained with Hoechst 33342 (ThermoFisher), followed by imaging with BZ-X800 (Keyence). Venus-positive cells were counted by QuPath 0.2.3 (34). After imaging, cells were harvested with supernatant and used for plaque assay. For RNA-seq analysis, 1.2 million cells (*n* = 4/group) were treated with RELMα (500 ng/mL) and infected 1 h later with IAV (WT). Twenty-four hours later, cells were recovered for total RNA isolation (lysis by Invitrogen TRIzol and RNA isolation by Promega, cat #Z3100). Samples were confirmed to have a 260/280 absorbance ratio of ~2.0. To ensure adequate IAV infection, qRT-PCR was performed to detect HA before 400 ng was sent to Novogene Inc. for RNA sequencing (20 million paired-end 150 bp reads, 30–50× depth).

### RNA-seq data processing and differential expression analysis

Raw data were processed for quality control using FastQC (35). Low-quality bases and adapter sequences were removed using trim_galore (36). High-quality trimmed reads were aligned to the mouse reference genome (GRCm39) using the STAR aligner (v2.7.9a) (37)with parameters –outFilterMismatchNmax 0 and –outFilterMultimapNmax 1 to discard reads mapped to multiple locations. QC data were summarized using MultiQC (38). Read counts were quantified using FeatureCounts (39). Differential expression analysis and visualizations were carried out in R (v4.2.0) (40) using the tidyverse (v2.0.0) (41), DESeq2 (v1.38.0) (42), and EnhancedVolcano (v1.16.0) (43) R packages. GO and KEGG enrichment analyses were carried out using AnnotationHub (v3.6.0) (44) and clusterProfiler (v4.6.0) (45) R packages.

### Pleural cells and lung epithelial cell isolation

Pleural cells were collected by washing the pleural cavity with 500 μL of ice-cold PBS two times using a 1,000 μL pipet tip. For lung epithelial cells, the mouse lung was perfused with 10 mL of ice-cold PBS and instilled with 800 μL of 1 U/mL Dispase (STEMCELL) and 100 μL of 1% low-melting point agarose (ThermoFisher). Lungs were incubated on ice for 2 min and incubated in 5 mL of HBSS solution (Lonza) at room temperature for 45 min. Lungs were minced with a scalpel in 5 mL of DMEM containing 25 mM HEPES (Corning), 1× penicillin-streptomycin (Gibco), and antimycotic solution (ThermoFisher). To generate a single-cell suspension of the lung cells, the solution was passed through a 70 μm then 40 μm nylon cell strainer. Filtered cells were spun down at 340 × *g* for 7 min and incubated with 3 mL of ACK lysis buffer (Gibco) to remove red blood cells. Pleural cells were incubated with biotinylated anti-MERTK antibody (BAF591, R&D System), and the lung single-cell suspension was incubated with anti-CD326 (EpCAM) microbeads (Miltenyi Biotec). Cells were sorted with MS or LS columns (Miltenyi Biotec) with the manufacturer’s instructions. Purity of lung epithelial cells was assessed by qRT-PCR for EpCAM or immunofluorescent staining of cytospins with antibodies to EpCAM (BioLegend).

### RNA isolation and qRT-PCR

RNA was isolated with the RNeasy Mini Kit (Qiagen), and qRT-PCR was run with GoTaq qPCR and RT-qPCR Systems (Promega) with the manufacturer’s instructions. Because HA cDNA was reverse transcribed from total RNA (vRNA, cRNA, and mRNA), the qPCR for the IAV HA segment measured both transcription and replication of the viral genome: *Epcam* forward: CGT GAG GAC CTA CTG GAT CAT, *Epcam* reverse: GTC CAC GTC GTC TTG TGT TTT; *Retnla* forward: CAA GGA ACT TCT TGC CAA TCC AG, *Retnla* reverse: CCA AGA TCC ACA GGC AAA GCC A; *HA* forward: GGA CAT GCT GCC GTT ACA C, *HA* reverse: AGC TCA GTG TCA TCA TTT GAA AGG T.

### Statistical analysis

All statistical analyses were conducted using GraphPad Prism software (v10.4; GraphPad Software, San Diego, CA). The data are presented as the mean ± standard error of the mean. We employed unpaired, two-tailed Student’s or Welch *t*-tests to assess statistical significance between two groups. For experiments involving more than two groups or multiple variables, a two-way analysis of variance was conducted, followed by Tukey’s post hoc test for multiple comparisons. Statistical significance was defined as *P ≤ 0.05, **P ≤ 0.01, ***P ≤ 0.001, and ****P ≤ 0.0001; ns means not significant. All experiments were repeated two to three times with *n* = 3–6 per group for each experiment apart from the RNA-seq analysis and epithelial cell sorting, which was done once with *n* = 4 per group. The corresponding figure legends provide exact *P* values and sample sizes (biological replicates).

## RESULTS

### IAV infection upregulates RELMα expression in the bronchoalveolar space and airway epithelium

**Fig 1:**
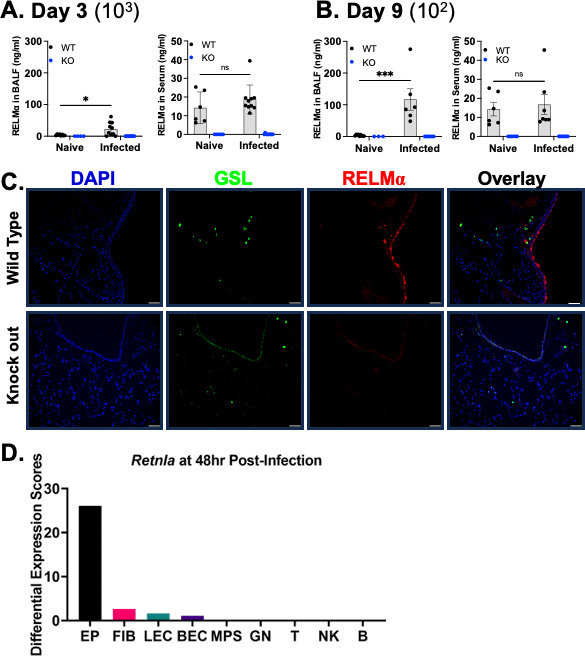
Influenza A virus infection induces epithelial cell-derived RELMα secretion. *Retnla*^+/+^ (WT) and *Retnla*^−/−^ (KO) mice were intranasally inoculated with PBS (naive), 10^2^ PFU (day 9), or 10^3^ PFU (day 3) of IAV (infected), followed by sacrifice at days 3 and 9. (**A and B**) RELMα in the BALF and serum was quantified by ELISA. (**C**) Lung sections of WT mice at 9 days post-infection were stained with anti-RELMα antibody and GSL and counterstained with DAPI (scale bar = 50µm). (**D**) RELMα gene expression was compared in lung single cells by using public single-cell RNA-seq data (GEO number: GSE109747). B, B cell; BEC, blood endothelial cell; Ep, epithelial; FIB, fibroblast; GN, granulocyte; LEC, lymphatic endothelial cell; NK, NK cell; MPS, mononuclear phagocyte system; T, T cell. Data for panels A and B are pooled from two independent experiments (*n* = 6–9 per group) and are presented as mean ± standard error of the mean. Statistical significance by two-way analysis of variance and Tukey’s post-test was defined as *P < 0.05 and ***P ≤ 0.001. ns, not significant.

RELMα expression is induced in the lung in Th2 cytokine responses, following injury, and in response to phagocytosis of apoptotic cells (46, 47). We hypothesized that the injury and inflammation induced by influenza virus may promote RELMα expression and could have functional implications for lung tissue responses. RELMα (*Retnla*)^+/+^ (WT) and RELMα^−/−^ (KO) mice were intranasally infected with A/California/04/2009 (H1N1) virus and sacrificed at 3 or 9 days post-infection, matching the peak of lung inflammation (day 3) and tissue resolution (day 9). 10^3^ PFU was used at day 3 post-infection, and 10^2^ PFU was used at day 9 post-infection to ensure mice survived to the tissue resolution stage. BALF and serum were collected from naive and infected mice to measure systemic and airway-specific RELMα. Infection resulted in an eightfold increase in RELMα protein in the BALF at day 3 post-infection (23 ± 7 ng/mL), which was further upregulated by 39-fold (117 ± 34 ng/mL) at 9 days post-infection compared to naive (3 ± 1 ng/mL) (Fig. 1A). In contrast, serum RELMα levels did not change with infection, suggesting infection site-specific effects (Fig. 1B). To spatially examine RELMα expression, IF staining of lung sections from WT and RELMα KO mice was performed for RELMα and *Griffonia simplicifolia* lectin (GSL), which binds to endothelial cells as well as macrophages (48–50) (Fig. 1C). The lung epithelium was RELMα positive, but the GSL-positive parenchymal macrophages did not express RELMα, indicating that IAV infection specifically upregulated RELMα expression in epithelial cells. A recent study described the heterogeneity in the response of lung cell populations following IAV infection, specifically using the mouse-adapted PR8 (A/Puerto Rico/8/34) influenza virus strain (51). Epithelial cells were found to contain more virus than any other cell types in the lung. We analyzed RELMα gene expression levels in heterogeneous lung cells 48 h post-IAV infection using publicly available single-cell RNA sequencing data from Steuerman et al. (GSE109747) from mice infected with PR8 virus (51, 52). We found that *Retnla* transcripts were differentially expressed between IAV-infected and control animals and that lung epithelial cells expressed the highest number of *Retnla* transcripts in the lung, supporting that RELMα upregulation by IAV infection is epithelial cell-specific (Fig. 1D).

### RELMα is expressed by IAV-infected epithelial cells and is associated with increased viral burden

**Fig 2:**
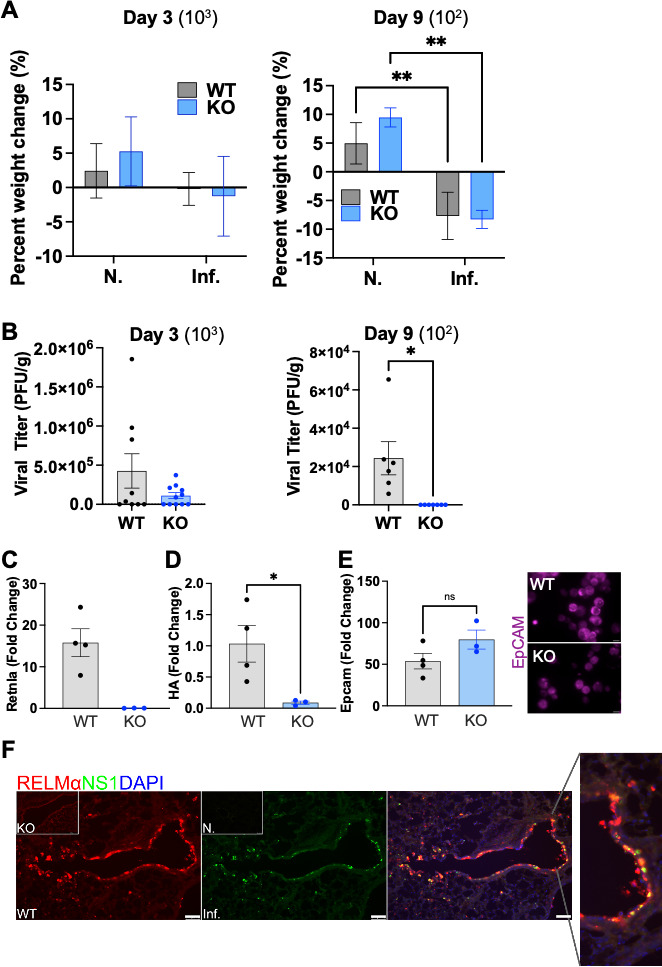
Reduced viral burden in RELMα-deficient lung epithelial cells. *Retnla*^+/+^ (WT) and *Retnla*^−/−^ (KO) mice were intranasally inoculated with PBS (naive) or 10^2^ PFU (day 9) or 10^3^ PFU (day 3) of IAV (infected), followed by sacrifice at 3 and 9 days post-infection. (**A**) Weight changes (%) at day 0 compared to day 3 or day 9 were measured. (**B**) Viral loads in the lung were quantified by plaque assay at day 3 and day 9. (**C–E**) Lung epithelial cells were isolated 7 days post-infection with 10^2^ PFU IAV, and *Retnla* and *HA* gene expression were measured by qRT-PCR as fold change over WT infected. Epithelial cell EpCAM was measured by qRT-PCR as a fold change over pleural macrophage EpCAM and visualized by immunofluorescent staining of cytocentrifuge preparations. (**F**) Lung sections from WT at 7 days post-infection with 10^2^ PFU were stained with antibodies against RELMα and NS1, then counterstained with DAPI. Inset controls include infected KO and naive (N) (scale bar = 100µm). Data, presented as mean ± standard error of the mean, are pooled from two independent experiments (*n* = 6–11 per group) for panels **A and B** and one experiment (*n* = 3–4 per group) for panels **C–E**. Statistical significance by two-way analysis of variance and Tukey’s post hoc test for panel A and unpaired Welch *t*-tests for panels** B–E **was defined as *P ≤ 0.05 and **P ≤ 0.01. ns, not significant.

The function of RELMα following IAV infection was investigated by comparing WT and RELMα KO mice. Influenza infection led to significant weight loss compared to PBS control mice at day 9 post-infection with 10^2^ PFU; however, there was no difference between WT and RELMα KO mice (Fig. 2A). In contrast, IAV plaque assays of lung tissue homogenate indicated that RELMα KO mice had lower IAV burdens, with no detectable live virus at day 9 post-infection, in contrast to WT mice (Fig. 2B). We determined epithelial cell-specific differences in RELMα expression and viral burden by magnetic-activated cell sorting column enrichment of EpCAM^+^ cells followed by quantitative RT-PCR. IAV infection upregulated *Retnla* expression in EpCAM^+^ cells compared to PBS control WT mice, while *Retnla* transcripts were not detected in RELMα KO EpCAM^+^ cells (Fig. 2C). IAV HA RNA was significantly higher in EpCAM^+^ cells from IAV-infected WT mice compared to RELMα KO mice (Fig. 2D), indicating that the difference between the lung viral burdens of wild-type and RELMα KO mice was epithelial cell-specific. No significant difference in *Epcam* RNA expression was observed between IAV-infected WT mice and RELMα KO mice (Fig. 2E). This finding was further confirmed by IF staining of lung sections for RELMα and NS1, a non-structural protein of IAV, which revealed RELMα^+^NS1^+^ lung epithelial cells that were absent in naive mice (N) and in infected KO mice (Fig. 2F). These results suggest that host RELMα expression is induced in IAV-infected lung epithelial cells with the detrimental outcome of increased viral burden.

### RELMα does not affect immune responses in the bronchoalveolar space

**Fig 3:**
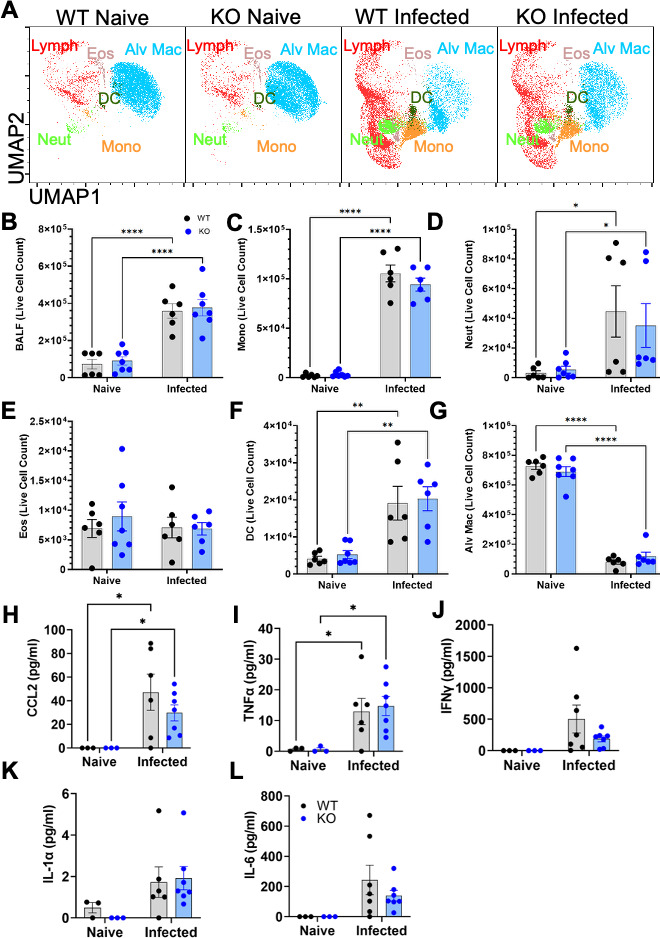
Equivalent IAV-induced lung immune responses in wild-type and RELMα-deficient mice. *Retnla*^+/+^ (WT) and *Retnla*^tDT/tDT^ (KO) mice were intranasally infected with 10^2^ PFU of IAV (infected) or PBS (naive), followed by sacrifice on day 9 post-infection. (**A**) Immune cell subsets in the BALF were identified according to the gating strategy shown in Fig. S1 and presented as UMAP plots. (**B**) Total live cell counts in the BALF. (**C–G**) Live cell counts of each immune cell subset in the BALF were enumerated by flow cytometry. (**H–L**) Concentrations of cytokines in the BALF were measured by cytometric bead array. Data, pooled from two independent experiments, are presented as mean ± standard error of the mean (*n* = 6–7 per group). Statistical significance by two-way analysis of variance and Tukey’s post hoc test was defined as *P < 0.05, **P ≤ 0.01, and ****P ≤ 0.0001. ns, not significant.

The lung is a structurally complex organ in which diverse cell types interact to defend the upper respiratory organs from insult. IAV infection of the lung increases immune cell infiltration and cytokine secretion, which are required for viral clearance. To test if RELMα affects the lung immune response to IAV, immune cell populations and cytokine levels were measured by flow cytometry and the Legendplex cytokine bead array of the BALF. Immune cell subsets were identified according to the gating strategy in Fig. S1, and UMAP plots were generated to evaluate immune cell subsets across the groups (Fig. 3A). IAV infection led to overall increased BAL cell numbers (Fig. 3B), with increases in the following subsets: monocyte, neutrophil, eosinophil, and DC (Fig. 3C through F), while alveolar macrophages were reduced (Fig. 3G). RELMα KO mice did not exhibit any differences in the numbers of immune cell subsets of the BALF. Cytokine concentrations in the BALF indicated infection-induced increases in proinflammatory cytokines CCL2 and TNFα, with a similar trend for IFNγ, IL-1α, and IL-6, which were equivalent in WT and RELMα KO mice (Fig. 3H through L). There were also no significant differences in BALF immune cell subsets or cytokines between wild-type and RELMα KO mice at day 3 post-infection (Fig. S2). Histological examination of IAV NS1 expression in lungs from WT and RELMα KO mice indicated higher IAV virus in the lungs of infected WT mice compared to infected KO mice. However, examination of H&E-stained lung sections from these mice revealed that infection-induced lung destruction, leukocyte infiltration, and vascular inflammation were not significantly different between WT and RELMα KO mice (Fig. S3). These results suggest that RELMα does not affect lung immune responses and pathology induced by IAV infection.

### Cell-specific deletion of RELMα in CC10^+^ bronchial epithelial cells is sufficient to reduce RELMα and IAV viral burden

**Fig 4:**
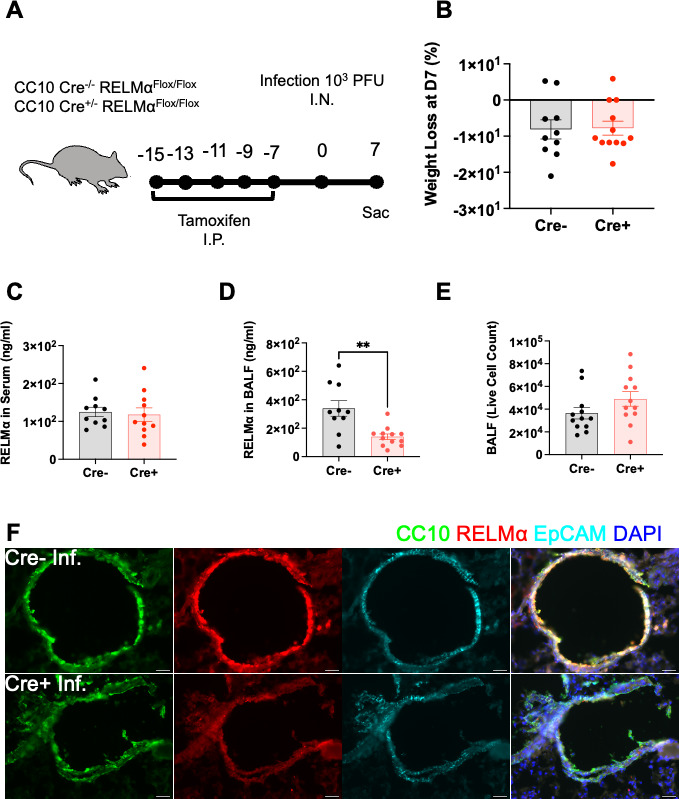
Epithelial cell-specific RELMα deletion following IAV infection. CC10 Cre^−/−^ (Cre−) or Cre^+/−^ (Cre+)/*Retnla*^Flox/Flox^ littermates were intranasally infected with 10^3^ PFU of IAV (infected), followed by sacrifice 7 days post-infection. (**A**) Schematic diagram of the experiment. (**B**) Infection-induced weight loss compared to pre-infection weight was calculated at 7 days post-infection. (**C and D**) RELMα in the BALF and serum was quantified by ELISA. (**E**) Total live cell counts in the BALF. (**F**) Lung sections of Cre− and Cre+ mice at 7 days post-infection were stained with antibodies against RELMα, CC10, and IAV and counterstained with DAPI (scale bar = 20µm). Results are pooled from two independent experiments and presented as mean ± standard error of the mean (*n* = 10–12 per group). Statistical significance by unpaired Welch *t*-tests was defined as **P ≤ 0.01.

Based on our findings that RELMα is expressed in the airway epithelium following IAV infection, we next investigated whether epithelial cell-intrinsic deletion of RELMα would be sufficient to confer increased protection from IAV infection. We utilized tamoxifen-inducible CC10 Cre mice to target club cells that line the bronchial epithelium (53). We generated CC10 Cre^+/−^ (Cre+) or CC10 Cre^−/−^ (Cre-)/RELMα^Flox/Flox^ littermates (RELMα^ΔCC10^ and RELMα^F/F^), such that tamoxifen injection would result in club cell-specific RELMα deletion (53), which was followed by IAV infection (Fig. 4A). Both RELMα^ΔCC10^ and RELMα^F/F^ mice exhibited equivalent weight loss following IAV infection (Fig. 4B) and no differences in serum RELMα levels (Fig. 4C). In contrast, RELMα concentration in the BALF was significantly reduced (Fig. 4D). Within the BALF of RELMα^ΔCC10^ and RELMα^F/F^ mice, there was no difference in total cell counts (Fig. 4E). Lung sections from IAV-infected RELMα^ΔCC10^ and RELMα^F/F^ mice were stained with antimurine CC10, EpCAM, and RELMα antibodies to confirm downregulation of RELMα in the airway (Fig. 4F). In infected RELMα^F/F^ mice, RELMα expression (red) was co-localized with EpCAM and CC10. In contrast, infected RELMα^ΔCC10^ mice had similar CC10 (green) and EpCAM (cyan) staining, but there was reduced, although not completely abrogated, RELMα expression.

**Fig 5:**
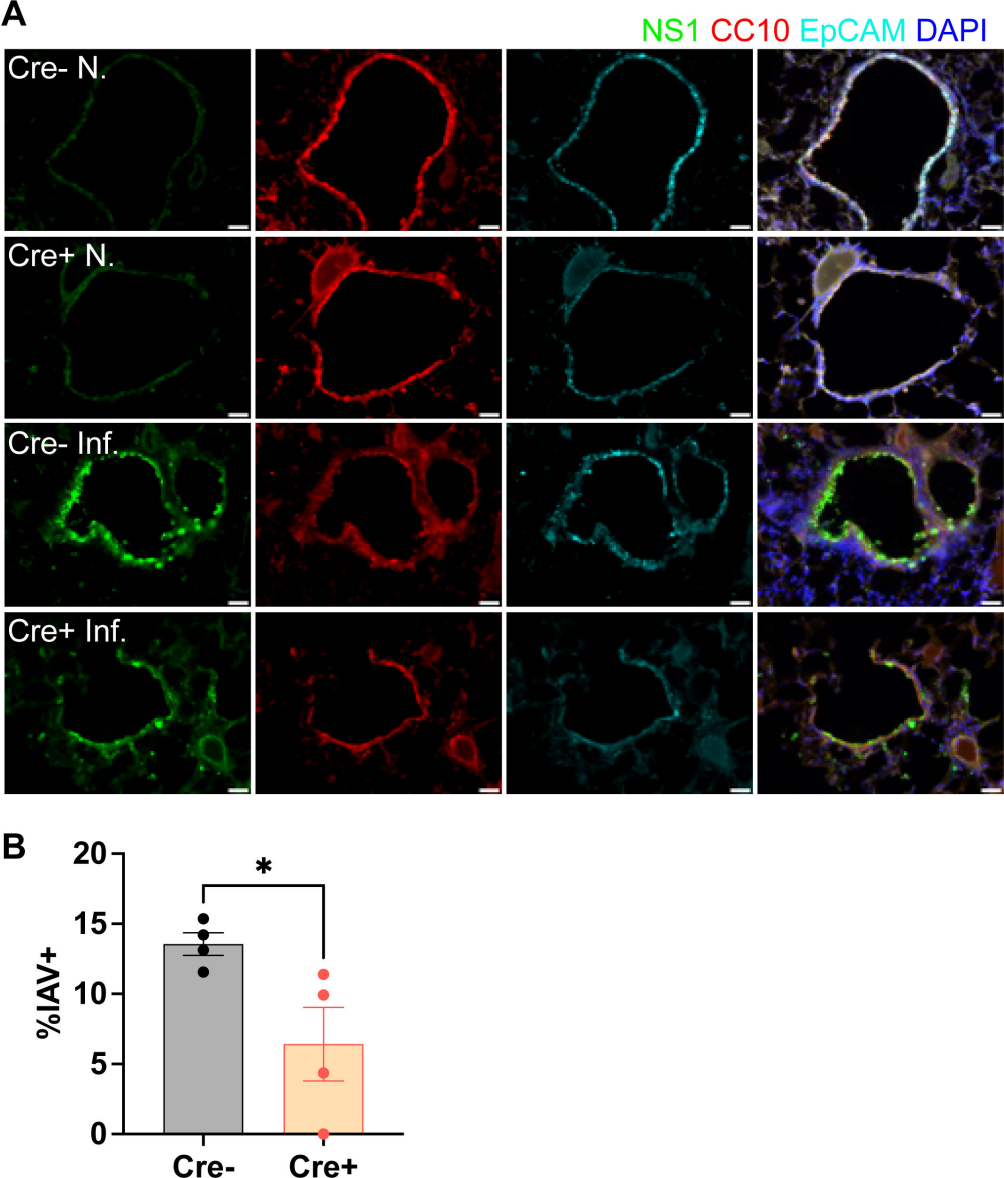
Reduced IAV-infected CC10 epithelial cells in CC10-specific RELMα-deficient mice. CC10 Cre^−/−^ (Cre−) or Cre^+/−^ (Cre+)/*Retnla*^Flox/Flox^ littermates were intranasally infected with PBS (naive) or 10^3^ PFU of IAV (infected), followed by sacrifice at day 7 post-infection. (**A**) Lung sections were stained with antibodies against EpCAM, CC10, and IAV (NS1), then counterstained with DAPI (scale bar = 20 µm). (**B**) IAV+ cells were counted in the infected groups and shown as a percentage of DAPI. Data are from one experiment and presented as mean ± standard error of the mean (*n* = 4 mice per infected group). Statistical significance by the unpaired Student *t*-test was defined as *P ≤ 0.05.

We next determined if CC10-specific RELMα deletion was sufficient to confer epithelial cell protection from IAV. Lungs from naive and IAV-infected mice were stained with antibodies against CC10, EpCAM, and IAV NS1 (Fig. 5A), which revealed that the bronchial epithelial cells of the airways of RELMα^ΔCC10^ had lower NS1 staining, suggesting lower IAV infection. This was validated by quantification of the IAV-infected cells, which exhibited significantly decreased NS1^+^ cell counts in the airways of RELMα^ΔCC10^ mice compared to RELMα^F/F^ mice (Fig. 5B). These data indicate that cell-intrinsic RELMα facilitates IAV infection of CC10^+^ epithelial cells.

### RELMα promotes IAV infection of mouse lung epithelial cells *in vitro*

**Fig 6:**
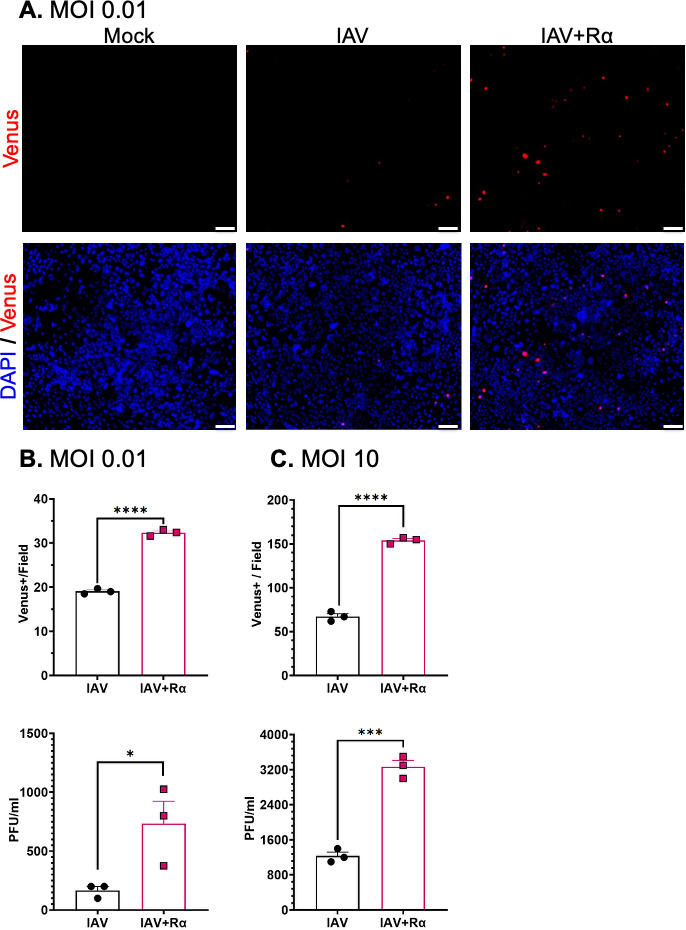
RELMα treatment increases IAV infection of murine lung epithelial cells. MLE-12 cells were pre-treated with PBS or RELMα (100 ng/mL) for 1 h, followed by infection with reporter IAV expressing Venus fluorescent protein at MOI = 0.01or 10. (**A**) Cells were treated with PBS or RELMα at MOI = 0.01, cultured for 72 h and imaged. (**B and C**) Viral burdens at MOI = 0.01 and MOI = 10 were quantified based on Venus reporter expression as a percentage of Hoechst-positive cells in 10 fields per well and by plaque assay of cell lysates that were collected at 72 h post-infection. Data are from three independent experiments, with three technical replicates per group, and are presented as mean ± standard error of the mean (*n* = 3 biological replicates per group). Statistical significance was defined by the unpaired Student *t*-test with *P ≤ 0.05, ***P ≤ 0.001, and ****P ≤ 0.0001.

The *in vivo* data utilizing whole-body and epithelial cell-specific RELMα KO mice in IAV infection support a paracrine function for IAV-induced RELMα in epithelial cells that benefits the virus and facilitates infection. Alternatively, the effects of RELMα may be indirect, through acting on another cell type that in turn acts on the epithelial cell. To determine if there was a direct function for RELMα, we utilized the murine lung epithelial cell line MLE-12, which is permissive for IAV infection (54). MLE-12 cells were pre-treated with 100 ng/mL of recombinant RELMα, which is in line with the concentrations measured in the BALF (see Fig. 1A) followed by infection with Venus reporter-expressing IAV to allowing live imaging of infected cells. Pre-treatment with RELMα led to significantly increased IAV virus (red) within the cells (Fig. 6A), which was quantified by Venus^+^DAPI^+^ counts and by plaque assays of the cell lysates at 72 h post-infection (Fig. 6B and C). These data suggest direct action of RELMα on lung epithelial cells that leads to increased permissiveness to IAV infection.

### RNA-seq analysis of a lung epithelial cell line identifies global gene expression and pathway changes upon IAV infection with RELMα treatment uniquely affecting metabolic genes

**Fig 7:**
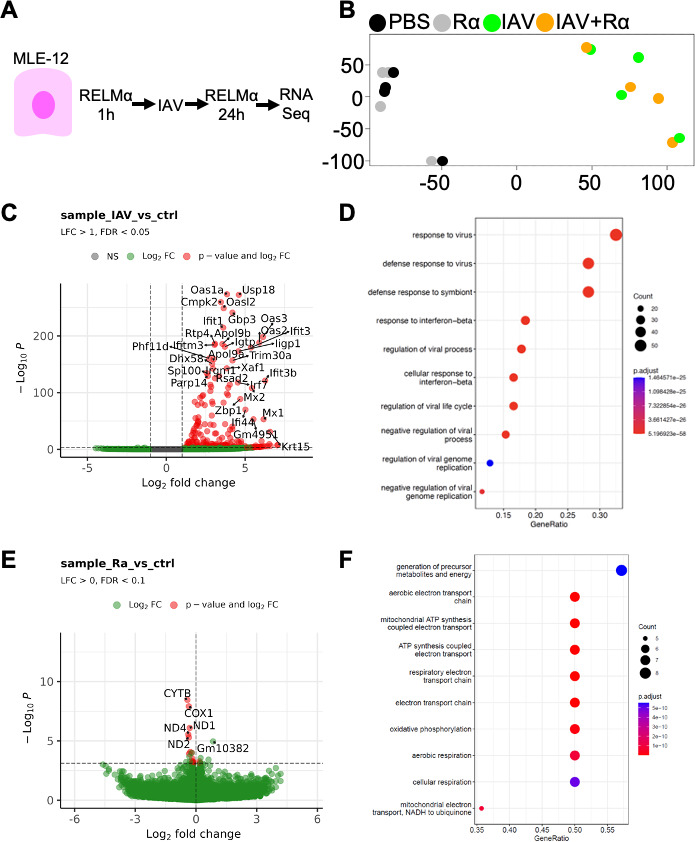
Gene expression changes by RELMα and IAV infection. MLE-12 cells were pre-treated with PBS or RELMα (500 ng/mL) for 1 h, followed by infection with IAV at MOI = 0.01 and recovery for RNA-seq analysis at 24 h. (**A**) Schematic of the experiment. (**B**) Principal component analysis plot. (**C**) Volcano plot of IAV-induced genes (log2 fold change >1, FDR <0.05). (**D**) IAV-induced basic pathways. (**E**) Volcano plot of DE genes (Ra vs Ctrl, log2 fold change >0, FDR <0.1). (**F**) Basic pathways are downregulated by RELMα (*P* < 0.1). Data are from one experiment, *n* = 4 per group.

To gain insights into the mechanism by which RELMα increases IAV infection, we performed RNA-seq on MLE-12 cells that were pre-treated with RELMα for 1 h and then infected with IAV for 24 h (Fig. 7A). We also performed RNA-seq on cells that were PBS treated and/or mock infected. We then compared each of these groups to each other, identifying differentially expressed genes (DEGs) as those genes with a log2 fold change (l2fc) of 1 or more with an adjusted *P* value less than 0.05 (Table S1). Principal component analysis revealed that the transcriptional profiles of RELMα-treated, uninfected cells and PBS-treated, uninfected cells were similar at 24 h post-infection (Fig. 7B, black and gray). These profiles were different from those of the RELMα-treated, infected cells and the PBS-treated, infected cells, which were similar to each other at that time point (Fig. 7B, green and orange). We first compared IAV-infected cells vs uninfected cells, with no RELMα treatment, and observed large numbers of upregulated DEG, indicating that the transcriptional response of MLE-12 cells to IAV infection was robust (Fig. 7C). IAV infection resulted in the upregulation of 192 genes. Interferon-stimulated genes such as IFIT1 and Mx1 were upregulated, demonstrating that the cells respond to IAV infection by producing type I interferons (Fig. 7C). The upregulated genes corresponded to GO pathways such as “response to virus,” “cellular response to interferon-beta,” and “regulation of viral life cycle” (Fig. 7D). No downregulated genes had an l2fc of 1 or more and an adjusted *P* value less than 0.05.

When we performed a comparison between RELMα-treated, uninfected cells and PBS-treated, uninfected cells to identify gene expression changes that occurred in response to RELMα treatment, there were no DE genes with an l2fc of 1 or more and an adjusted *P* value less than 0.05. When we adjusted our cutoff to an adjusted *P* value less than 0.1 and absolute l2fc > 0, we identified 14 downregulated genes and two upregulated genes (Fig. 7E). The downregulated genes were enriched in cellular metabolism-associated pathways such as “generation of precursor metabolites and energy,” “electron transport chain,” and “oxidative phosphorylation” (Fig. 7F). No DE genes were identified when RELMα-treated, IAV-infected cells were compared to PBS-treated, IAV-infected cells, suggesting that RELMα-induced transcriptomic changes occurred earlier than 24 h after RELMα treatment and/or that transcriptional responses to IAV infection outweighed any specific effects of RELMα. Together, these data provide a useful transcriptional profile of MLE-12 cells following IAV infection and identify modest effects of RELMα on transcription that were related to changes in cell metabolic activity.

## DISCUSSION

In this study, we demonstrate that RELMα is induced by IAV infection and plays a non-protective role in promoting viral infection of epithelial cells. RELMα is a pleiotropic protein that is expressed by diverse cell types such as epithelial cells and immune cells and is upregulated by different stimuli (17, 18, 55, 56). RELMα expression in epithelial cells is mostly IL-4Rα dependent, but it is also expressed in the absence of type II inflammation (30, 32), consistent with our study demonstrating upregulation in IAV infection. A previous study showed that OSM, a gp130 cytokine, elevated the expression of RELMα in airway epithelial cells (31). OSM was upregulated by H3N2 IAV infection in human nasal epithelium. It is possible that RELMα upregulation by IAV infection in lung epithelial cells may be mediated by OSM through the gp130 receptor pathway. Gp130 cytokines are also referred to as the IL-6 family of cytokines. Although not to the same extent as OSM, IL-6 also increased the expression of RELMα in airway epithelial cells (31). OSM was not measured in this study; however, we observe that IL-6 was elevated with IAV infection. IAV-induced IL-6 could therefore have contributed to RELMα expression. Immunofluorescent staining for IAV NS1 protein and RELMα indicated co-staining of RELMα with IAV-infected cells, suggesting that infection itself could induce RELMα, although the specific molecular pathway and signals involved are unknown.

When RELMα is expressed in the Th2 cytokine environment in the lung, it downregulates inflammation and accelerates the repair of damaged tissue (22, 24, 25, 29, 57). Here, instead, RELMα promoted influenza virus infection of epithelial cells without affecting the immune response. Previous studies showed that RELMα activates the phosphatidylinositol 3-kinase (PI3K)/AKT and ERK pathways to promote proliferation of mesenchymal stem cells (56, 58). RELMα also elevated ERK phosphorylation in mouse lung fibroblasts, which was required for inhibition of apoptosis induced by TNFα and cycloheximide (59). It is possible that RELMα induces proliferation and survival of the host cells that will become viral reservoirs. On the other hand, the bivalent role of PI3K in influenza virus infection and host cell defense was identified in another study (60), where IAV titers were significantly reduced upon treatment with the PI3K inhibitor, wortmannin, increasing the accumulation of viruses on the surface of cells, indicating that PI3K activation is required for virus uptake. In a related study, treatment of A549 lung epithelial cells with a broad-range tyrosine kinase inhibitor decreased IAV entry (61). IAV was shown to induce the activity of receptor tyrosine kinases such as epidermal growth factor receptor (EGFR). Sialic acid and EGFR were found to co-localize non-randomly on the surface of A549 cells (62). Their co-localization coupled receptor binding to EGFR activation, which stimulates IAV uptake by endocytosis. This suggests that viruses exploit host antiviral components to facilitate their infection and survival in host cells. RELMα’s ability to support IAV infection in epithelial cells may therefore be due to the combined effects on receptor tyrosine kinase signaling, increased proliferation, and apoptosis inhibition.

Utilizing epithelial cell-specific RELMα-deficient mice and IAV infection of a murine epithelial cell line (MLE-12), we show that the negative impact of RELMα function in supporting IAV infection is directly through effects on epithelial cells. Transcriptomic analysis of the MLE-12 cells indicated robust gene expression following IAV infection, notably significant gene upregulation with no gene downregulation, indicating that IAV infection of MLE cells provides a suitable *in vitro* model that leads to metabolically and transcriptionally active cells. It is hoped that this data set, comprising 192 upregulated genes, can provide a useful framework to study epithelial cell-specific pathways in IAV infection. Surprisingly, despite functional differences in viral burdens in response to RELMα, there were very few or no differentially expressed genes in response to RELMα. This may have been due to timing, given that RNA-seq was performed only at one time point of 24 h and the fact that there was no detectable RELMα transcript in MLE-12 cells, indicating that cell-intrinsic expression of RELMα did not occur in these *in vitro* assays. Another caveat is that MLE-12 cells require estradiol and hydrocortisone for culture, which could inhibit immune responses. Last, given that virus infection alone caused such a dramatic increase in gene expression, any effects of RELMα treatment could have been masked. Intriguingly, the only genes that were differentially expressed upon RELMα treatment were associated with cell metabolism and mitochondrial function, including NADH dehydrogenase (ND1, 2, and 4), cytochrome B, and cytochrome oxidase (COX1). It is possible that this RELMα-induced change in metabolic activity results in inhibiting antiviral gene expression or apoptosis, leading to more viral replication in the surviving cells (63). Future studies investigating the human RELM proteins, RELMβ and resistin, would provide valuable clinical relevance in the potential function for these proteins in viral infection of epithelial cells.

In conclusion, our study uncovers an unexpected role for the immune protein RELMα in subverting the host response to influenza infection through direct effects on epithelial cells. Targeting RELMα or downstream pathways may provide promising strategies to mitigate viral subversion. Future studies to pinpoint these mechanisms are warranted.

## Data Availability

The transcriptomic data were submitted to the NCBI GEO database under accession number GSE268747.

## SUPPLEMENTAL MATERIAL

Figure S1 jvi.02225-25-s0001.tif Flow cytometry gating strategy.

Figure S2 jvi.02225-25-s0002.tif Equivalent IAV-induced lung immune responses in wild-type and RELMα-deficient mice at day 3 and day 7 post-infection.

Figure S3 jvi.02225-25-s0003.tif Equivalent IAV-induced lung pathology in wild-type and RELMα-deficient mice at day 9 post-infection.

Supplemental legends jvi.02225-25-s0004.docx Descriptive legends for Fig. S1 to S3 and Table S1.

Table S1 jvi.02225-25-s0005.xls Transcriptomic data.

ASM does not own the copyrights to Supplemental Material that may be linked to, or accessed through, an article. The authors have granted ASM a non-exclusive, world-wide license to publish the Supplemental Material files. Please contact the corresponding author directly for reuse.
