# Supplementary material for: RELMα establishes a permissive environment for influenza virus infection through direct effects on lung epithelial cells
Source: J Virol. 2026 Jul 16;100(8):e02225-25. doi: 10.1128/jvi.02225-25 (PMC13483350; doi:10.1128/jvi.02225-25)
Supplement: Supplementary file 1 [file jvi.02225-25-s0004.docx]

**SUPPLEMENTAL MATERIAL**

**SUPPLEMENTAL TABLE 1:** List of DEG and Gene Ontology pathways comparing IAV vs Control (tabs 1, 3), RELMα vs Control (tabs 2, 4).

**FIGURE S1:** **Flow Cytometry Gating Strategy**

**FIGURE S2: Equivalent IAV-induced lung immune responses in wild-type and RELMα-deficient mice at Day 3 and Day 7 post-infection.** *Retnla*^+/+^ (WT) and *Retnla*^tdT/tdT^ (KO) mice were intranasally infected with 10^2^ PFU of IAV (Infected) or PBS (Naive), followed by sacrifice on day 3 or 7 post infection. **(A)** Total live cell counts in the BALF were enumerated. **(B-F)** Live cell counts of each immune cell subset in the BALF were enumerated by flow cytometry. **(G-N)** Concentrations of cytokines in the BALF were measured by cytometric bead array. Data is pooled from 2 independent experiments and presented as mean ± SEM (n = 6-7 per group). Statistical significance by two-way ANOVA and Tukey’s post-hoc test was defined as *p<0.05; **p≤0.01; **p≤0.001; ns not significant.

**FIGURE S3: Equivalent IAV-induced lung pathology in wild-type and RELMα-deficient mice at Day 9 post-infection.** *Retnla*^+/+^ (WT) and *Retnla*^tdT/tdT^ (KO) mice were intranasally infected with 10^2^ PFU of IAV (Infected) or PBS (Naive), followed by sacrifice on day 9 post infection. Lung tissue sections were prepared for **(A)** Hematoxylin and Eosin (H&E) staining and **(B)** blinded pathology scoring.
